# The Impact of COVID-19 on the Educational Process: The Role of the School Principal

**DOI:** 10.1177/00220574211032588

**Published:** 2021-07-19

**Authors:** Charalampous Constantia, Papademetriou Christos, Reppa Glykeria, Athanasoula-Reppa Anastasia, Voulgari Aikaterini

**Affiliations:** 1 Neapolis University Pafos, Cyprus

**Keywords:** COVID-19, distance education, school principal

## Abstract

In recent years, the role of the leader in the effective operation of the school has been vastly debated in the international educational community. Through a historical study of educational leadership, this research discovered that the position of the leader is constantly being reshaped and adapted to the current social, cultural, and economic circumstances. During the last year, due to the coronavirus disease 2019 pandemic, the educational leadership has had to be reshaped worldwide. The aim of this study is to investigate the issues that have arisen from the aforementioned situation, as well as to try to figure out how a school’s principal might apply the basic principles of educational leadership, in a period of crisis. This investigation was focused on the use of the qualitative method. The study included 88 teachers and 5 principals from Cyprus, as well as teachers and parents. Based on the findings of the study, we discovered that the challenges faced by school principals and teachers are primarily linked to alienation, marginalization, time management, improving bureaucracy, problems with technical equipment and distance learning programs. Solution to these obstacles seemed to be the: Empathy, teamwork, and decentralization of the educational system, which are all promoted by the principal, who occasionally has additional authority.

## Introduction

In recent decades, research on educational leadership has exploded. Previously, the word “management” was used to express the essence of the term administration, but, in nowdays, has a restrictive meaning and is synonymous with enforcement, homogeneity, and behavior of members of organizations, while the definition of administration is associated with forecasting, preparation, organization, communication, and control in the organization (Connoly et al., 2019).

This term has a broader meaning than the term administration. The term management is most often associated with the regular activity of organizations’ functionality, while the term administration refers to the execution of a particular project or the enforcement of a policy (Ali & Abdala, 2017). Both words are translated in Greek under the word «διοίκηση», a fact that sometimes causes confusion and misunderstandings.

However, let us attempt to clarify the terminology. Management is more of a holistic administration mechanism that leads to efficiency improvement by satisfying the needs and wishes of the parents. In other words, it is an arrangement focused on the relations between school and society (Brown & Oplatka, 2012).

A school principal is an individual who is designated as an administrative representative by a higher authority to promote specific interests by coordinating and managing organizations, corporations, or educational units that have a specific function (Bredeson, 2000).

However, the word “leader” has a different meaning. In general, we can assume that a leader should have those characteristics, such as the following:

(a) the creation and transmission of a common vision, (b) the promotion of distributed work, (c) the creation of a collective culture, (d) the development and understanding of staff, (e) the creation of productive relationships with parents and the community, (f) the ability to handle proper communication tactics (Day & Sammons, 2016).

Furthermore, leadership is defined by principles and inventions, and it seeks to affect individuals and communities. It deals with vision, strategic challenges, change, outcomes, and people. Leadership, in general, has the ability to inspire people to work harder than they expect. It appears that we might call it art, in which the producer, mediator, and actor is the leader. Leadership is twofold. On one hand, it is a process that contributes to the accomplishment of pre-determined goals while on the other hand, it builds the school’s vision based on the leader’s personal and professional values (Rapti, 2013).

Although a manager is in charge of management and administration, a leader is in charge of leadership. In short, the leader is the school’s emblem, both for the students and for society as a whole. He or she is the individual who sets the levels, being in charge of the school’s events and directly responsible for the improvements and promotion of knowledge and appropriate behavior (Rapti, 2013).

## Educational Leadership

The above circumstances have had a major impact on the field of education. Nevertheless, educational leadership is peculiar, as Everard et al. (2004) point out, for two reasons. The school is an administrative entity with strong bureaucratic features, as it is a decentralized service established by the applicable legislation. Furthermore, the school is a social organization with a significant presence in the local and broader communities, from which it attracts and exerts pressure.

The behavior of a leader–principal is one of the most critical factors in building successful schools (Oaltay & Karada, 2016). However, in the field of education, the principal is responsible for organizing school life, ensuring compliance with laws and ministry of education circulars, issuing official orders, and carrying out teacher association decisions (Edo et al., 2019). The principal is the individual who plans and implements curriculum growth as well as enhances the operation of staff and students by mobilizing and directing them toward the school’s objectives (Çoğaltay & Karadağ, 2016). As a result, one might argue that educational leadership is dynamic enough to resemble a mix of traits. The principal is the one who has to be very familiar with all of these traits so that he or she can incorporate them in the work setting to effectively adopt them (Leithwood et al., 2008).

As a result, the principal is also responsible for implementing educational innovations. It must, however, be well equipped with a variety of features to be successful. According to Charalampous and Papademetriou (2019), the principal is the school’s chief who serves as the leader of a social community and he or she is also a designated employee of the educational authorities. As a leader, he or she strives to meet the expectations of the people he or she serves, namely the teachers and students, while as an appointed employee, he or she strives to meet the expectations of his or her educational pyramid’s hierarchical superiors (A. Reppa, 2008). It also encourages creativity, solves problems, and interacts with parents and the local community.

According to Improving the Quality of Education for All (IQEA), principals should: (a) emphasize school improvement, primarily through the quality of learning of all students, (b) encourage all members of the school community to participate in the improvement process, (c) promote in the school the idea that its actors should seize opportunities for change, and (d) create appropriate learning environments, which will promote collaboration and give strength to the participants (Beresford et al., 2003).

Consequently, the principal is more than just a manager of the school’s affairs. This model must be replaced with the model of the principal–supporter–leader, who takes initiative, is flexible and innovative, and formulates internal educational policy (Charalampous & Papademetriou, 2020).

Thus, the leader must turn schools into environments where teachers and students can participate in activities that assist them in being successful and coping with the issues and challenges they face (Ainscow, 1998). A successful leader must also consider topics like professionalism, accountability, ethics, and efficient school administration practices. This type of leadership is founded on intelligence, beliefs, and abilities (Begley, 2001).

To summarize, no one can deny that the principal in a school plays a critical role in its performance and effectiveness (IQEA, 2004). In addition, the effectiveness of the school is also dependent on the institution itself; whether or not, it has the requisite conditions to provide equal opportunities to all students (Slee & Weiner, 2001).

## Coronavirus Disease 2019 and Its Side Effects in Educational Leadership

The coronavirus disease 2019 (COVID-19) pandemic has had a major impact on our everyday lives over the last year (Azorin, 2020). To regulate the spread of this disease, stringent restrictions have been enforced all over the world (Harris & Jones, 2020). This fact has affected many facets of human life, including schools, which are being called upon to restructure their educational roles and processes (Zhao, 2020).

As is well recognized, educational leadership is a dependent mechanism that adapts and must adapt to social, economic, and cultural factors (Alhouti, 2020). In this scenario, the manager must function remotely (Harris, 2020) in a timely, accurate, targeted, and efficient manner (Netolicky, 2020). However, as predicted, he or she is confronted with various unprecedented issues, which he or she must address immediately and distantly. According to Harris and Jones (2020), the absence of interpersonal relationships, in their typical form, face-to-face contact between teachers students, parents, school management, and the broader school community, are among the biggest problems that the principal is called upon to face during the pandemic. In addition, the principal must take into consideration and deal with the feelings of both teachers and pupils. According to the most recent research in Cyprus (Haztilia Drotarova et al., 2020), the fear is the most prevalent emotion, which is followed by confusion and anxiety, brought about by the lockdown’s ambiguity. Also, according to Ravitch (2020), pandemics have exacerbated feelings of marginalization among some students.

At the same time, the principal’s position has become too bureaucratic, as it has to deal with the ministry of education circulars, health protocols, and regulations. Unfortunately, the principal is forced to cope with all these, without further explanatory direction from educational stakeholders (Harris & Jones, 2020).

According to recent reports (Harris & Jones, 2020), principals should be educated in crisis management problems such as the COVID 19 pandemic, as well as in issues related to the use of distance education technology. Of course, we could not ignore the fundamental values of educational leadership during the lockdown: the leader’s strong vision and the professional development (Leithwood et al., 2008).

## Emotional Intelligence and Emotional Leadership

If the school principal used emotional intelligence as a key lever, the school’s attempt to provide inclusive education could be boosted even further. Emotional intelligence, according to Bar-On (2000), is a mixture of social and emotional abilities, adaptive skills, and personality traits. Emotional intelligence, according to Aldiabat (2019), is a vital factor in leading a good leader since it encompasses self-knowledge, self-control, social awareness, and relationship management. In the same vein, Doe et al. (2015) argue that emotional intelligence is a key trait of a good leader, in other words, the ability to self-regulate and appreciate other people’s feelings, as well as the use of empathy as a management tool.

A principal with emotional intelligence can have a positive impact on the whole school community’s culture. Moore (2009) claims that when a school principal inspires both, teachers and students, by recruiting their emotional intelligence, then he or she can achieve pedagogical goals more efficiently.

During this pandemic, the principal’s mobilization of emotional intelligence has vanished from the school administration, as there is no direct interpersonal interaction due to the lockdown. Even human interaction is reliant on technology. Unfortunately, both teachers and students, as well as school administration, may lack the requisite technical skills to deal with the distance education generated by the pandemic.

## Materials and Methods

The focus of this research was the investigation of the difficulties that a principal had to cope with, because of the lockdown and the compulsory distance education. The research questions of the present study are the following:

Research Question 1 (RQ1): Which difficulties do teachers face due to compulsory distance education?
Research Question 2 (RQ2): What difficulties does the principal face due to compulsory distance education?
Research Question 3 (RQ3): How can the principal of a school cope with the problems that arise due to compulsory distance education?

Because of the nature of the issue, which was linked to the subjective opinions of those directly involved in the study, a qualitative research approach was chosen as the most suitable. As a result, study methods such as unstructured interviews, participatory observation, and focus groups were used. The survey was conducted in four Cypriot cities (Limassol, Paphos, Larnaca, and Nicosia) with the participation of 93 people, including teachers from five public secondary schools. Specifically, there were 88 teachers, 42 primary school teachers, 46 secondary school teachers, and the 5 principals of the 5 schools in which the research was conducted.

The sample of the research was derived from two levels of education (secondary and primary) because of one important distinction between the two levels. During the lockdown, secondary education students, as well as primary students (fifth and sixth grades), attended distance learning courses. The remaining primary school students, on the other hand, received regular asynchronous guidance and homework from their teachers.

The research was conducted after a written completed consent form by the principals of the schools and all the participants. At the same time, the research team ensured the anonymity and confidentiality of the data. The data of the research were documented, transcribed, coded, and analyzed, which enabled the researchers to identify information about the topic under study from the interview and extract the key thematic units.

Due to the lockdown, the interviews were conducted using electronic video conferencing programs with open cameras and microphones, allowing us to concentrate on the gestures of the participants as well as their speech and reaction during the interviews.

## Results

The current study was initiated by a focus group, which took place from September 2020 to February 2021, after participants had experienced the detrimental effects of the COVID-19 pandemic. The focus group was held in the afternoons and outside of school buildings, to prevent negative feelings such as feeling uncomfortable that some teachers might not be present at their workplace school. Furthermore, due to the individual schedules of each participant, it was difficult to find a common hour and hold this meeting in the morning. The emphasis of the discussion was on the issues that teachers face during the COVID-19 pandemic’s lockdown phase, which led to distance learning. The following viewpoints were expressed:

*In the last year, the teaching profession has undergone significant changes. This, of course, has an effect on the school principal’s job. We assumed that the teacher instructs in a classroom with his students in attendance. Now even that has been overturned* (Primary Education Teacher).

*That’s right . . . But from this point forward some problems rise up considering the role of the teacher and therefore the role of the principal* (Primary Education Teacher).

*The fact that the teachers and the administrative staff do not meet in person exemplifies the issue. They don’t talk, they don’t spend time together, and they may not even know each other very well. How would they be able to work together on school tasks and solve student problems if something so basic and obvious is missing?* (Teacher of Secondary Education)

*Indeed, colleagues . . . You have right . . . We have become into ordinary bureaucrats. We also hold meetings over the internet, which very often have no apparent cause. There is no essential contact and communication* (Primary Education Teacher).

*Email, email, email, email, email, email, email, email, email, email, email, email It’s time to stop . . . We collect orders on a regular basis and carry them out like a robot* (Teacher of Secondary Education).

*Without any doubt, for us the teachers, but mostly for the principals, there is more bureaucratic work* (Teacher of Secondary Education).

Examining the views that emerged during the focus group observation, we concluded that one of the main problems caused during the lockdown was the increase in bureaucratic work, which exacerbated the already depressing atmosphere generated by the lockdown. We then proceeded to individual interviews, which aimed at in-depth investigation of the problems that might have arisen due to the current situation. However, let’s examine the perspectives of the participants:

*Our limited knowledge of computers and software programs is a big concern. We find it challenging to teach remotely because we do not know how to manage microphones, loudspeakers, or the teaching software itself . . . You can realize that this puts both teachers and school principals under a lot of pressure* (Primary School Teacher).

*What you have said is related to the degree to which students understand what we teach, but also to the degree to which they are watching us. We cannot know if they are truly watching us whether they are playing a game for example. The microphones and cameras are off* (Secondary Education Teacher).

*It is also worth noting that children do not concentrate enough on distance learning* (Primary Education Teacher).

*I have a difficulty in managing time. Sometimes I complete my lesson earlier than I expected, while other times I do not have enough time to complete it* (Primary School Teacher).

The views of the participants led to the conclusion that a major concern that arises during distance learning is the proper management of teaching time, which is negatively affected by technical issues that occur during the lesson and are difficult to be solved, since teachers are unfamiliar with the programs and devices that are used to deliver the course. In addition, there seems to be a lack of focus and attention among the students. Unfortunately, the problems that arise during distant learning do not end here. On the contrary, they spread both in the social and psychological field, influencing both the work of teachers and the work of school principals. Specifically, the participants expressed the following sentiments:

*We are all becoming anti-social. We have to be in front of a computer constantly* (Primary Education Principal).

*Neither we, nor the principal can sense what the kids are going through. We have no idea what they are thinking or how they are feeling about the situation* (Secondary Education Teacher).

*We cannot talk to the principal or our colleagues about the problems we are facing. We hide our emotions. We are not as comfortable with each other as when we were at school* (Primary Education Teacher).

*Let’s not forget that there are children who might be marginalized, either because they do not have the necessary equipment or internet connection, in order to be able to attend online lessons, or because they experience some other marginalizing situations that become more intense with distance learning. For example, their mother tongue may not be Greek. They may also have some learning disabilities and need additional guidelines to comprehend what the teacher is saying* (Secondary Education Principal).

*Another issue that the teachers and the school management face is that the children have become anti-social. After the lockdown the students are less willing to cooperate, so the learning process became much more boring compared to the days before the pandemic* (Secondary Education Teacher).

*Children aren’t inspired anymore. What can I say to motivate them to work? Compete, and engage in activities? Inform them that they are going on an excursion? That’s what we’d tell them in the past. Now, even if you tell them, they know it’s a lie* (Secondary Education Principal).

*We have done so many events before. We celebrated anniversaries, we organized conferences, we hosted award ceremonies . . . Nowadays, we are scared if more than 15 children are gathered in one place . . . Even these are too many* (Primary Education Principal).

The participants’ views, both teachers’ and principals’, lead us to the conclusion that the lockdown and consequently distance learning have led children to social distancing, affecting their emotional world.

While conducting this qualitative research, we have asked the principal of a high school to permit us to attend a distant meeting of the teacher association (since one of the researchers belonged to the teaching staff of the school). It would be important to examine the following:

*The meeting has begun. Within five minutes time, most of the teachers entered the virtual chat room. One by one they said: Hello, I’m . . . ». . Gradually they began to give me the impression that they did not just want to greet their colleagues but did so formally to show the principal that they were present, since he did not see them. After about 30 minutes, the principal started talking about the issues scheduled for the meeting. From that moment on, the principal simply announced the topics of the meeting without being interrupted. No one knew if anyone disagreed or agreed. No one could see the expressions and reactions of the meeting participants, since the cameras were off. Then he started asking the teachers, according to their specialty, if they had accomplished the tasks assigned to them. At this point, the disagreements started since some teachers said that they had not received the email of the meetings with the orders, others said that they had not understood exactly what was asked of them, while some others said that they would have preferred to have discussed with the principal how to deal with a specific problem, rather than simply being called upon to carry out the orders of the Ministry.*

Examining the above observation, it appears that even during a distant meeting of a teacher association, problems arise, as some participants may be reluctant to take active action in the meeting, while others state that the live meeting is more efficient.

Moreover, it would be significant to investigate the participants’ perspectives on the principal’s position and, more precisely, the management style during a lockdown.

*The management of the school has changed dramatically. Suddenly, everything started to function electronically by everyday emails. The worst thing is that we had to read all these messages very carefully since they contained instructions for the school administration and the conduct of the lesson. Face-to-face communication is no longer possible, we must be extremely cautious when sending emails, in order to avoid any misunderstandings* (Secondary Education Principal).

*The role of the principal has changed. Cause of the pandemic, he became a bureaucrat who merely carried out orders from the Ministry. Moreover, these orders are changing daily because the Ministry is updating them day to day. You literally spending too much time and you get lost in orders and circulars without producing any substantial work. You’re just the messenger* (Primary Education Principal).

*During the pandemic and the lockdown, the principal is obliged to tell his subordinates how and when to carry out orders. The human factor has disappeared. The principal no longer sees his subordinates as unique entities. He shuffles laws and circulars to find out which category they belong to and manage if they are eligible for a facilitation. He has to put them in groups. One in vulnerable group, the other in parents of children under 15 years old and so on. This definitely leads to a great waste of time.* (Primary Education Principal).

*Because of this situation, it seems that the human relations that the principal once had with the teachers have been lost. He cannot immediately find a solution to everyday issues and he is forced to spend time on phone-calls and mobile messages to clarify the rights of the teachers based on the Covid-19 law* (Secondary Education Principal).

*The management team is often forced to track down and call parents to inform them that their child is required to stay at home due to contact with a coronavirus case. It takes a long time to complete this task. Ι then set out to decide which students will return to school and when they will do so. Occasionally, I have the feeling that I am a doctor rather than a school principal* (Secondary Education Principal).

*During lockdown, we asked for hours parents and students if they have an internet connection at home, if they have a tablet, a computer, a telephone, if they can download the program to do a lesson . . . I believe we have lost the point. Everything else was put on hold so we could focus solely on the pandemic’s issues. This is a disaster. I feel that I am not doing my job well* (Primary Education Principal).

*During the pandemic, there were times that some students had to return in school, such as primary school students and the third year of high school. In high schools this situation has caused huge problems. The management teams had to see which teachers were returning, if they managed to be on duty during the breaks according to the program, if they were also keep teaching in the classes that operate remotely. All this created many questions and dissatisfactions on the part of the teachers, which each principal tried for many hours to resolve* (Secondary Education Principal).

*Every day there were problems related to the management of the electronic platform through which distance learning was conducted. Thus, he recruited the teacher with computer skills to prepare some instructions regarding the use of Teams platform. The principal was the one who had the responsibility to organize seminars and trainings, in order to solve these problems* (Primary Education Principal).

The above observation’s data analysis indicates that the principal faces a key problem: the lack of effective communication between the teachers’ association and the principal, since the teachers simply received instructions from the principal, without any essential interaction. The principal has become a bureaucrat, who is lost in orders, circulars, and health protocols. Therefore, in a lockdown period he or she functions as a school principal and a representative of the Ministry of Education, rather than as a leader carrying a vision that he or she co-decides with the Teacher Union.

At this point, it is important to mention that the data analysis proves convergence of views among principals and teachers regarding the problems that the principal encounters in a lockdown period. Similarly, primary and secondary education teachers’ views seem to agree.

The problems that have arisen in schools, as a result of the COVID-19 pandemic and the compulsory lockdown, are numerous and varied. Nonetheless, finding a solution to this problem is important. At this stage, it is worth looking into the perspectives of the research participants on potential solutions to the problem.

*Certainly our options are limited in times of pandemic, certainly the problems are many and we cannot solve them easily, but in a good mood and through collaboration we can achieve a lot. However, collaboration among teachers, but also between the management and the teachers, is something that the principal should promote* (Primary Education Teacher).

*The lesson must become more interactive. To do this, teachers must be properly guided by the principal. We need to find ways, depending on the nature of each course, to involve students in the learning process. We can, for example, divide them into groups, since we have through the conferencing software the possibility to create 2-3 different chat rooms within our online class. These groups can, with the help of the teacher, discuss issues related to the course* (Primary Education Principal).

*The school principal must organize online seminars, which will aim to train teachers in distant learning issues. He may request this from a computer teacher or from an external partner* (Secondary Education Principal).

*Online seminars could be organized for teachers and parents related to adolescent psychology. These seminars could provide the teacher with practical advice in order to enhance better communication with the pupils* (Secondary Education Teacher).

*The principal should develop emotional intelligence so that he/she can understand the feelings of both teachers and students in times of crisis, such as during a lockdown* (Primary Education Principal).

*During lockdown period, it would be better for principals to have more authorities and the system to be less centralized, in order to avoid unnecessary bureaucracy, which really troubles both teachers and school principals* (Secondary Education Principal).

Concluding this research, it would be very significant to refer to the participants’ suggestions for dealing with and avoiding the difficulties caused by the coronavirus pandemic. According to the participants, the lesson should become more interactive, and there should be more intense cooperation among the members of the school community. In addition, the principal should organize seminars addressed to teachers, parents and students, regarding both the use of electronic software for distance learning and topics related to child and adolescent psychology. Furthermore, during world crisis, such as in the case of COVID-19 pandemic, a partial decentralization of the educational system could take place. By this way, the principal would have more authorities and therefore less beaurocracy. Finally, the principal’s role could be enforced by the development of emotional intelligence, so that he or she gain the ability to understand others’ feelings. However, in order to achieve this, principals need to be trained through seminars and self-help exercises on a) knowledge and management of emotional intelligence, but also on b) mindfulness, in order to focus on the issue that concerns them each time (G. Reppa, 2020a, 2020b).

## Discussion

The analysis of the data of the present research showed that during the lockdown and therefore the distant learning, both the learning process and the role of the school principal have changed. The results of this research are in accordance with Zhao (2020) research, who proved that the crisis we have all experienced has strongly influenced teaching and learning process and therefore the role of the school principal.

In particular, the research data analysis has led to the conclusion that among the problems that arise due to the lockdown and distant teaching is the increase of bureaucratic work, which exacerbates the already depressing climate and affects negatively the time management. Another difficulty is the technical problems that arise during the lesson and are difficult to be solved. This happened because teachers are not familiar with the electronic platform that is used to conduct distant learning.

In addition, there seems to be social distance among the school community. On one hand, the students faced difficulties in concentrating during the online lesson while on the other hand, there is a lack of effective communication between the teacher association and the principal. This comes in line with Harris and Jones (2020) research, who supported that there is no interaction between teachers and principal since the teachers simply received instructions from the principal, without any discussion. The principal has become a bureaucrat who gets lost in orders, circulars and health protocols. He or she does not behave as a leader, with a common vision with the teacher association, but as a school administrator and a representative of the Ministry of Education.

According to the research findings, the role of the principal is very crucial in problem-solving. Both teachers and principals can cope up with the aforementioned difficulties in various ways. First, the principal should promote collaboration among the teachers’ association. Second, he or she can underline the importance of motivation by encouraging the teachers to develop interactive lessons. Moreover, he or she should organize seminars addressed to teachers, parents, and students regarding the use of the computer distance platform and adolescent psychology. Another suggestion that emerges from the result analysis of the present research is the partial decentralization of the educational system, which can give the principal even occasionally more flexibility, so he or she can have better and more effective time management. Finally, as the participants proposed, the enhancement of the principal’s emotional intelligence could be a very helpful “weapon” in overcoming the crisis.

Despite the significant results of this research, there are also some limitations. In future research, the methodology should be a mixed research method design, including both qualitative and quantitative data, to enhance the triangulation of the research results. Furthermore, the sample could be bigger by recruiting students and teachers, from all school grades, as participants. By doing that, the research will have more objective and representative results which can be further generalized. Concluding, future research must be conducted in investigating the role of the school principal in overcoming the educational crisis.
